# Identification of Human SARS-CoV-2 Monoclonal Antibodies from Convalescent Patients Using EBV Immortalization

**DOI:** 10.3390/antib10030026

**Published:** 2021-07-05

**Authors:** Rut Valgardsdottir, Irene Cattaneo, Gavino Napolitano, Annibale Raglio, Orietta Spinelli, Silvia Salmoiraghi, Concetta Castilletti, Daniele Lapa, Maria Rosaria Capobianchi, Claudio Farina, Josee Golay

**Affiliations:** 1 Center of Cellular Therapy “G. Lanzani”, Division of Hematology, ASST Papa Giovanni XXIII, 24127 Bergamo, Italy; rutvalg@gmail.com (R.V.); icattaneo@asst-pg23.it (I.C.); ospinelli@asst-pg23.it (O.S.); ssalmoiraghi@fondazionefrom.it (S.S.); 2 Division of Microbiology and Virology, ASST Papa Giovanni XXIII, 24127 Bergamo, Italy; gnapolitano@asst-pg23.it (G.N.); araglio@asst-pg23.it (A.R.); cfarina@asst-pg23.it (C.F.); 3 Fondazione per la Ricerca Ospedale Maggiore, 24127 Bergamo, Italy; 4 Virology Laboratory, INMI-IRCCS “L. Spallanzani”, 00149 Roma, Italy; concetta.castilletti@inmi.it (C.C.); daniele.lapa@inmi.it (D.L.); maria.capobianchi@inmi.it (M.R.C.)

**Keywords:** SARS-CoV-2, monoclonal antibody, EBV

## Abstract

We report the isolation of two human IgG1k monoclonal antibodies (mAbs) directed against the SARS-CoV-2 spike protein. These mAbs were isolated from two donors who had recovered from COVID-19 infection during the first pandemic peak in the Lombardy region of Italy, the first European and initially most affected region in March 2020. We used the method of EBV immortalization of purified memory B cells and supernatant screening with a spike S1/2 assay for mAb isolation. This method allowed rapid isolation of clones, with one donor showing about 7% of clones positive against spike protein, whereas the other donor did not produce positive clones out of 91 tested. RNA was extracted from positive clones 39–47 days post-EBV infection, allowing VH and VL sequencing. The same clones were sequenced again after a further 100 days in culture, showing that no mutation had taken place during in vitro expansion. The B cell clones could be expanded in culture for more than 4 months after EBV immortalization and secreted the antibodies stably during that time, allowing to purify mg quantities of each mAb for functional assays without generating recombinant proteins. Unfortunately, neither mAb had significant neutralizing activity in a virus infection assay with several different SARS-CoV-2 isolates. The antibody sequences are made freely available.

## 1. Introduction

The COVID-19 pandemic, first detected in Wuhan in late 2019, reached Europe as well as other countries in Asia and America by January 2020. Subsequently, many mutants have emerged with greater infectivity compared to the original strain. The dramatic spread of the disease led to the rapid mobilization of many laboratories and clinical units to find treatments, including the search for neutralizing monoclonal antibodies (mAbs). These can be isolated by several different techniques and have shown efficacy in many other infectious diseases [1,2,3,4]. Methods include isolation from human VH-VL libraries, direct antibody sequencing from memory or antigen-specific B cells, purified from individuals post-infection or post-vaccination, expansion in vitro of B cell clones, as well as more classical mAb production from immunized animals followed by humanization [1,5]. The application of these techniques has allowed the identification of many neutralizing anti-SARS-Cov-2 mAbs, several of which have reached the clinic (reviewed in the works of [6,7]).

Our region was the first and one of the most badly hit among European countries. Bergamo, more specifically, has become unfortunately world-famous for the difficulty in dealing with the dramatically increased death rate, estimated to be about 15 daily deaths/100,000 inhabitants locally during the peak of the epidemic, and causing the death of up to 0.15% of the population in the epicenter during the first wave [8], a problem later also experienced elsewhere. The pandemic rapidly led to the conversion to COVID-19 wards of most units of the main town hospital of about 800-bed capacity. During March 2020, up to 70–80% of beds in the entire hospital were occupied by COVID-19 patients [9,10]. The intensive care unit (ICU) passed from 46 to 100 beds, 88 of which were dedicated to COVID-19 patients [9,10]. Many doctors, nurses, and other personnel also succumbed to the infection during the first months of the pandemic [9]. Despite these extreme difficulties, essential activities for very ill and urgent patients, such as oncology patients, were maintained, with the great effort and dedication of all personnel involved [9,10]. In our laboratory, during the first very strict lockdown, which lasted 8 weeks from 8 March to 4 May 2020, only life-saving clinical laboratory activities by a restricted number of staff were allowed, all other workers being strictly confined at home at all times, except to purchase food or for health issues.

In this context, since our unit had previous expertise in culturing B cells, their immortalization with Epstein Barr virus (EBV) and therapeutic monoclonal antibodies (mAbs), as well as the availability in-house of some of the first tests for detection of anti-SARS-CoV-2 antibodies, we set out to try and isolate new mAbs from local personnel who had recovered from COVID-19, as soon as the complete lockdown was lifted, allowing non-essential activities to be resumed. We used a simple EBV immortalization technique, starting from peripheral blood from convalescent COVID-19 patients. This method allowed the relatively rapid isolation of two B cell clones expressing IgG1 antibodies against SARS-Cov-2 Spike protein in sufficient quantity for purification and functional assays without using genetic engineering techniques. MAb production by selected B cell clones was quite stable over time. Our results are discussed in the context of mAb isolation for COVID-19 and other infectious diseases.

## 2. Materials and Methods

### 2.1. Donors, Cell Purification, and Infection with EBV

The marmoset B95-8 cell line producing EBV has been described previously [11] and was expanded in RPMI1640 medium supplemented with glutamine (Euroclone, Pero, Italy), gentamycin (Fisiopharma, Palomonte, Italy), and 10% fetal bovine serum (FBS) (Euroclone). It was grown to confluence and supernatant collected 3–5 days after confluence and used fresh after 0.8 µM filtering (Millipore, Cork, Ireland).

The study was approved by the Hospital Ethical Committee in accordance with the Declaration of Helsinki of 1975. Whole blood was collected in EDTA vacutainers (BD Biosciences, San Jose, CA, USA) from donors after informed consent.

Mononuclear cells (PBMC) were isolated from peripheral blood by standard Ficoll Hypaque gradient centrifugation (Cedarlane, Burlington, ON, Canada). Switched memory B cells were further purified by magnetic selection using the Memory B Cell Isolation Kit (Miltenyi Biotec, Bergisch Gladbach, Germany) according to the manufacturer’s instructions.

Memory B cells were resuspended in 1 mL complete RPMI advanced medium (Gibco, Thermo Fisher Scientific, Waltham, MA) containing 2.5 µg/mL ODN2006 CpG (Invivogen, Toulouse, France). A total of 1 mL B95-8 filtered supernatant was added and cells incubated for 5 h at 37 °C, 5% CO_2_. Cells were then diluted to either 50 or 250 B cells/mL in a complete medium containing 1% B95.8 culture supernatant, 2 × 10^5^/mL autologous irradiated PBMC, 2.5 µg/mL ODN2006 CpG, 50 IU rhIL2 (Proleukin, Novartis Pharma Gmbh, Nurnberg, Germany), and 500 ng/mL cyclosporine A (Novartis Pharma Stein AG, Stein, Switzerland), and 200 µL of the solution was distributed in flat-bottom 96-well plates. Half the volume of each well was replaced once a week with fresh medium containing ODN2006 CpG, rhIL-2, and cyclosporine A until clones could be expanded, after which only rhIL-2 was supplemented to the cultures. Some infected cells were plated in 6-well plates to generate a pool of EBV-LCL.

### 2.2. SARS-CoV-2 Antibody Tests

Anti-SARS-Cov-2 spike antibodies were quantified in donors’ serum and culture supernatants using the LIAISON^®^ SARS-CoV-2 S1/S2 IgG kit on a LIAISON^®^ XL platforms (DiaSorin S.p.A, Saluggia, Italy) according to the manufacturer’s instructions. Some supernatants were also tested with the Elecsys^®^ Anti-SARS-Cov-2 Nucleocapsid Protein Kit (Roche Diagnostics, Basel, Switzerland).

### 2.3. IgG Characterization and Sequencing

Immunoglobulin isotype was analyzed by staining cells with FITC-labeled anti-IgG, IgA, and kappa, and PE-labeled lambda specific antibodies (BD Biosciences) and flow cytometry. IgG1 subclass was determined on clone supernatants using the Pro-Detect™ Rapid Human Antibody Isotyping Assay Kit (Thermo Fisher Scientific).

RNA was purified from the positive clones and cDNA synthesized by reverse transcription with Superscript IV VILO Master Mix (Thermo Fisher Scientific), followed by amplification of the variable region of IgH, Igκ and Igλ transcripts by PCR with primers recognizing family-specific heavy chain (HC) leader sequences (VH1-VH6) [12] or light chain (LC) leader sequences (Vκ1/2, Vκ3-Vκ4, and Vλ1-Vλ8) [13] and reverse primers complementary to Ig constant regions (Cγ/Cκ/Cʎ) using GoTaq DNA polymerase (Promega, Madison, WI, USA).

Positive PCR was purified with Wizard SV Gel and PCR Clean-Up System (Promega) and sequenced (Eurofins Genomics, Ebersberg Germany). The sequences were aligned with germline genes and analyzed using the IMGT software.

### 2.4. Cell Expansion in Serum-Free Medium for Antibody Purification

For antibody purification, B cell clones were expanded in X-VIVO 15 medium (Lonza, Verviers, Belgium). MAbs were purified from the pooled supernatants using Protein A Ultralink Resin (Thermo Fisher Scientific) and elution in Gentle Ab/Ag Elution Buffer (Thermo Fisher Scientific), according to the manufacturer’s instructions. Purified mAbs were dialyzed overnight against PBS and sterilized by 0.2 µM filtration (Meran, Istanbul, Turkey).

### 2.5. SARS-Cov-2 Neutralization Test

The purified mAbs were tested for neutralization using three virus strains: Clade G VR10734 (S-D614G mutant, hCoV-19/Italy/LOM-INMI-10734/2020; GISAID Accession ID: EPI_ISL_568579), Clade O (2019-nCoV/Italy-INMI1; GISAID accession ID EPI_ISL_410546), and VOC 202012/01 (hCoV-19/Italy/CAM-INMI-118/2020; GISAID Accession ID: EPI_ISL_736997; Lineage B1.1.7) with serial dilution of purified antibody from the two positive clones (0.5 mg/mL to 0.5 ng/mL) according to published procedures [14]. Briefly, mAbs were diluted in serum-free medium and titrated in duplicate in ten-fold dilutions. Equal volumes of 100 TCID_50_/well of each virus strain and mAbs dilutions were mixed and incubated at 37 °C for 30′ and then added to sub-confluent Vero E6 cells and incubated at 37 °C and 5% CO_2_. Seroneutralization was measured after 72 h incubation, based on the cytopathic effect observed in an inverted microscope [14]. Serum from the National Institute for Biological Standards and Control, U.K. (NIBSC) with known neutralization titer was used as a reference in the microneutralization assay (Research reagent for anti-SARS-CoV-2 Ab NIBSC code 20/130). To standardize assay procedures, positive control samples showing high (1:160) and low (1:40) neutralizing activity and negative control samples were included in each microneutralization assay session. The highest serum dilution inhibiting the cytopathic effect indicates the neutralization titer.

## 3. Results

### 3.1. Isolation of Two EBV-Immortalized B Cell Clones Secreting Anti-SARS-CoV-2 IgG1 Monoclonal Antibodies

Whole blood was collected from two local volunteers within 4 months after their infection with SARS-CoV-2. Donor A was a 27-year-old female, confirmed to be serologically positive for anti-spike S1/S2 in two separate serological tests performed 1–3 months after asymptomatic infection (65 and 73 AU/mL with the Spike S1/S2 IgG Kit from DiaSorin). Her blood was collected for the EBV infection experiment within 4 months of infection, based on likely infection not earlier than the 1st week of February 2020, positive IgG antibody test in early May, and blood collection on 4 June 2020. Donor B was a 50-year-old male who had experienced moderate symptoms, in particular, a 2-week-long fever, PCR-confirmed diagnosis on 9 March, and a strongly positive antibody test (150 AU/mL) several weeks later. His blood was collected for EBV infection on 2 July 2020, about 4 months after infection. We can infer that blood samples from donors A and B were collected 2–4 and 4 months after infection, respectively.

**Figure 1:**
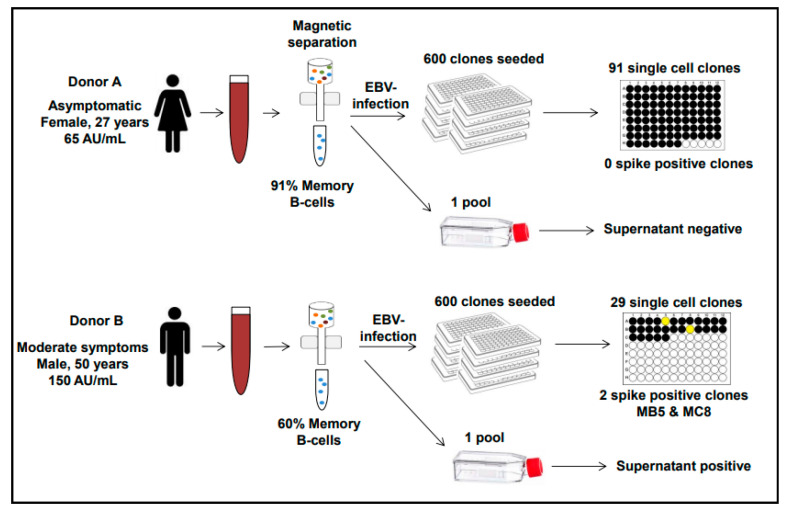
Experimental flow diagram. The experimental setup to isolate anti-SARS-CoV-2 antibodies is summarized for each donor.

Switched memory B cells were purified from PBMC by magnetic separation, infected with EBV, and plated at limiting dilution to generate single immortalized B cell clones. The remaining cells were plated in a 6-well plate to generate a pool of immortalized B lymphoblastoid cells (EBV-LCL). Cell clones and pools were inspected weekly, growing wells expanded, supernatants collected, and viable cells cryopreserved as soon as possible. A total of 600 wells were initially plated for each donor (for a total of 1200 wells). A total of 91 clones from donor A and 29 from donor B started to grow after 2–3 weeks and could be expanded and tested for their secretion of anti-SARS-Cov-2 spike protein IgG antibody production. Unfortunately, about 70% of the growing clones from donor B were lost to inadvertent microbial contamination. The experimental flow is summarized in Figure 1.

A total of 2 out of 29 clones (6.9%) from donor B tested positive in the spike S1/S2 IgG test (71.5 and 72.7 AU/mL, respectively) at day 39 and 47 of culture post-EBV infection (MC8 and MB5, respectively) and were further expanded. None of the 91 clones from donor A tested positive (0%) (Figure 1 and Table 1). The supernatant of donor B pool also tested weakly positive in the same test (14.9 AU/mL), whereas the donor A pool remained negative, in line with the presence of more frequent B cell clones producing anti-spike antibody in donor B than in donor A (Figure 1 and Table 1).

A smaller number of clones and the cell pools from both donors were also tested for anti-SARS CoV-2 nucleocapsid antibodies when this test became available in our hospital. No nucleocapsid-positive clones were identified (0/18 and 0/29, for donors A and B, respectively), although the pool supernatant from donor B was found to be positive also for the nucleocapsid protein (cutoff index of 29.9) (Table 1).

**Figure 2:**
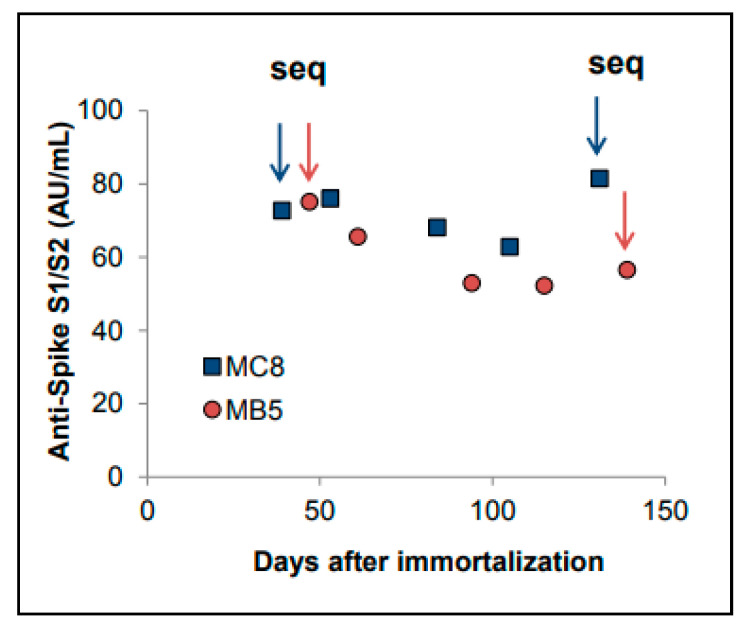
Stability of expression of IgG by MB5 and MC8 clones during culture. The anti-spike antibody levels in the supernatants of the 2 clones were measured at different times during long-term culture. The RNA was extracted at the 2 time points indicated by arrows and VH-VL regions amplified and sequenced. Identical sequences were obtained from early and late cultures of MB5 and MC8.

The positive clones were expanded in culture, aliquots of cells were cryopreserved at different times, and supernatants were collected and stored. We observed that antibody production was constant over time; stable antibody production was detected in the supernatant of cells expanded in a complete medium containing rhIL-2 for up to 130–140 days continuous culture (>50AU/mL) (Figure 2). From day 66–68, the MB5 and MC8 clones were adapted and expanded in serum-free medium to facilitate antibody purification.

### 3.2. PCR Amplification and Sequencing of IgG1 Clones

**Figure 3:**
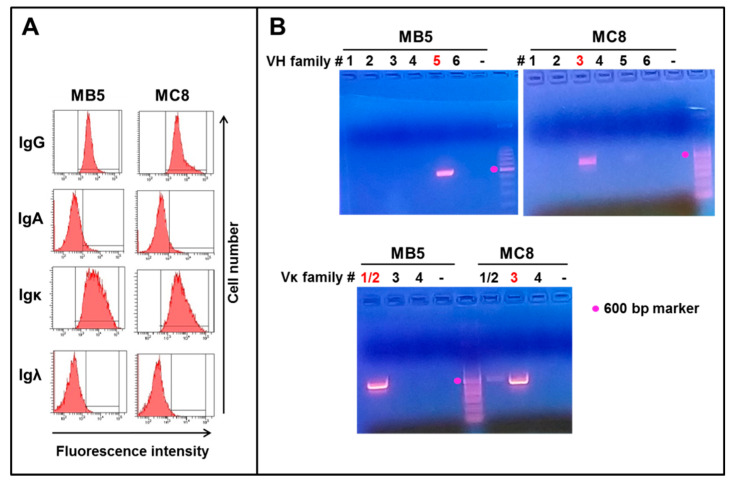
Identification of IgG isotypes and VH and Vk families for the MB5 and MC8 clones. (**A**) The IgGk isotypes of the 2 clones were identified by flow cytometry. (**B**) RNA from each clone was amplified by PCR using forward primers specific for the different families of heavy chain leader sequences (VH1 to VH6 families, upper panels) or κ light chain leader sequences (Vκ1/2, Vκ3 or Vκ4 families, lower panel) and common reverse primers. The numbers above each lane identify the VH or Vk family numbers that were probed with specific primer sets. The VH or Vk families that resulted in positive PCR bands are shown in red.

The two mAbs MB5 and MC8 were identified by flow cytometry and rapid testing as IgG1kappa (Figure 3A and data not shown). RNA was extracted from each clone at days 39–47 post immortalization, and heavy and light chain genes were amplified by PCR using sets of 6 and 10 primers recognizing the major VH and VL leader sequence families, respectively, and common Ig Cγ/κ/ʎ constant regions. The MB5 clone was identified by PCR to belong to the VH5 and Vk1/2 families, whereas the MC8 clone showed positive PCR signals with primers specific for the VH3 and Vk3 families (Figure 3B, V family primers resulting in an amplified band are indicated in red).

**Figure 4:**
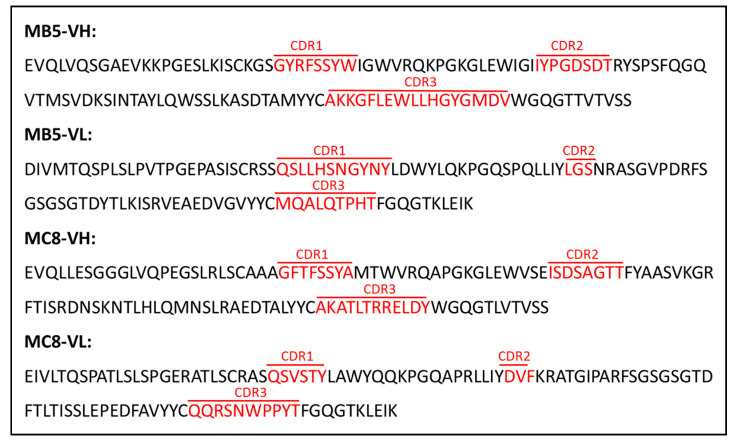
Deduced amino acid sequences of variable domains of the MB5 and MC8 clones. The sequences of the variable regions of heavy and light chains (VH and VL) of MB5 and MC8 clones are shown. CDR1-3 regions are indicated in red.

The MB5 and MC8 VH and Vκ regions products were amplified, purified and sequenced. The V regions nucleotide sequences of the two clones are shown in Supplementary Figure S1. The deduced amino acid sequences are shown in Figure 4.

The sequence analysis identified likely germline genes and revealed that the VH sequences of the MB5 and MC8 clones are 96.18% and 94.79% identical to the closest germline sequences (IGHV5-51*01 and IGHV3-23*01, respectively) (Table 2). The JH sequences are more divergent, as expected, whereas Vk sequences show 99.66% and 98.11% identity with the closest germline genes (IGKV2-28*01 and IGKV3-11*01, respectively) (Table 2).

RNA extraction and sequencing were repeated on the same clones expanded up to day 131–139. The sequences obtained were identical to the first isolates, suggesting a lack of significant ongoing mutational events in the variable regions of the IgGs and clonal stability of the MB5 and MC8 clones within the time frame analyzed.

### 3.3. Purification and Functional Assays

Culture supernatants from the expanded MB5 and MC8 clones were collected, pooled, and purified on protein A. The purified mAbs showed a spike S1/S2 binding activity of 103 and 81 AU/mL at a 1:2 dilution in the diasorin test. They were then tested by microneutralization assay, using three different Italian SARS-CoV-2 viral isolates belonging to Clades G and O and the lineage B1.1.7 U.K. [14]. The two anti-spike mAbs did not show any measurable neutralizing activity even at the highest concentration tested of 0.5 mg/mL. In contrast, the two control sera with high and low neutralization titer tested in parallel had a neutralizing activity at a 1:160 and 1:40 dilution, respectively.

## 4. Discussion

We describe here the isolation and sequences of two mAbs specific for the SARS-CoV-2 spike protein derived from a convalescent COVID-19 patient. The results show that the identification and isolation of antigen-specific mAbs can be achieved from immune donors relatively rapidly, with a low throughput method and in difficult conditions immediately following an unexpected and dramatic pandemic peak [9,10]. The isolated EBV-immortalized clones were expanded in vitro and could produce enough purified mAbs for functional analysis without further genetic engineering for the production of recombinant mAbs in HEK293 or CHO cell lines as would usually be performed in therapeutic mAb development. Sufficient supernatant and freezing of cells for initial screening purposes were achieved at days 39 and 47 post-EBV infection for the two positive clones, respectively. A total of 1200 wells were plated, and screening of all clones (about 120), including slow-growing ones, was achieved within 10 weeks of immortalization. The method of isolation of polyclonal EBV-immortalized cell lines for producing therapeutic human antibodies was first described in the late 1970s [15,16]. Adding a cloning step led to the identification, production, and use of therapeutic human anti-rhesus D mAbs [11,17]. Modified techniques for isolating memory and/or antigen-specific B cells, improving EBV infection rate, and expansion of single clones have been later developed and optimized, most prominently by Antonio Lanzavecchia’s group. Indeed, human mAbs against a wide variety of infectious agents, including SARS-CoV, influenza virus, Ebola, HIV, etc., have been relatively easily identified and isolated [1,2,3,4,18,19,20].

In our conditions, we observed that plating 10–50 infected memory B cells/well in 96-well plates was the best concentration to allow outgrowth of cells in about 1 out of 3–10 wells, which is a reasonable guarantee of clonality. Indeed, subsequent immunophenotyping and sequencing confirmed the clonality of the expanded cells. Unfortunately, most clones from donor B were lost to contamination, and the two clones isolated did not produce neutralizing antibodies, as hoped for. It is interesting to note that the percentage of positive clones paralleled the positivity of the supernatants from the pools of EBV-LCL from the same donors. Donor B had a positive signal in his EBV-LCL pool and showed a positivity rate of nearly 7% in the screening of clones (2/29), whereas donor A had no positive signal from the EBV-LCL pool supernatant and no clone was identified (0/91), suggesting a clonal frequency below 1%. These data suggest possible strategies of screening first the EBV-LCL pools, which grow within 2–3 weeks after EBV infection and subsequently concentrating the analysis on clones from donors showing positive signals in pools. The results are also in line with previous reports investigating the frequency of virus-specific memory IgG^+^ B cells, which were found to vary from 0.1% to 25% in different donors or when probing different antigens or viruses [21,22]. This frequency was also reported to increase up to 5–200-fold following boost vaccination or at peak post-natural infection [21,22]. Similar data have been published for SARS-CoV-2 specific memory B cells and plasmablasts [23,24].

In order to facilitate the screening of clones, several groups have shown that it is possible to measure their frequency in different donors and also purify these cells using tagged recombinant proteins to facilitate specific mAb isolation [5,25]. We attempted to do this using the first commercially available tagged SARS-CoV-2 spike or nucleocapsid recombinant proteins, but we could not identify a specific signal (data not shown). The reason for this failure is not clear but may in part be related to the fact that initial commercially available reagents were not optimal and that natural spike protein is a trimeric protein [26,27].

Other strategies exist to isolate human antigen-specific mAbs from immunized donors, such as the direct sequencing of VH and VL domains by RNA sequencing (scRNA seq) of purified single antigen-specific memory B cells or antibody-secreting plasma cells [1,25,28,29,30,31,32,33]. These, however, are time-consuming, require subcloning of VH/VL sequences in plasmid vectors, transfection in mammalian cells, and purification of recombinant mAbs before functional assays can be performed. These methods, therefore, require more specialized laboratories with already established high throughput techniques [5,25,32,33].

The EBV immortalization method used here allowed us to expand cells continuously, producing antigen-specific antibodies in the supernatant for up to 140 days post-infection and 100 days after positive clone identification. Milligrams amount of mAbs could also be purified. Furthermore, VH and VL sequencing at early and late points of the culture showed that no significant somatic mutations were taking place in the two isolated clones during this time frame. The stability of mAb production by EBV-immortalized B cells is somewhat controversial, and few data are available in literature on this point. Some groups have reported ongoing mutations or unstable expression during the time [34]. However, these observations may result from the fact that in some cases, cell lines rather than clones were studied or that other immortalization methods were used [34]. Our observed stable expression of mAbs by the EBV clones is in agreement with the results of Lanzavecchia’s group [1]. This method, therefore, allows performing relatively rapidly a number of functional assays, such as affinity measurements as well as neutralization assays using naturally human B cell-produced antibodies, expected to carry all the natural post-translational modifications of antibodies, such as glycosylation [35]. In addition, the method required few specialized components and was achieved in a standard cell culture lab and during a critical period of the explosive pandemic. We also had the advantage of the availability of local donors (from the hospital and laboratory staff), allowing fast identification of donors with high serum titers of anti-SARS-CoV-2 antibodies.

Donor B also had detectable anti-nucleocapsid antibodies in the EBV-LCL pool, but no anti-nucleocapsid clones were identified in the few that could be analyzed. Interestingly, donor B had experienced COVID-19 symptoms, in particular fever, for about 2 weeks, and had high titer anti-spike antibodies in his serum (150 UA/mL), whereas donor A had not even realized she had been infected until she tested positive for the anti-SARS-CoV-2 antibodies (65–68 AU/mL) in the context of a screening of all hospital staff in May 2020. We cannot at this point know whether the high mAb levels and high frequency of anti-spike B cells in donor B are a result of symptomatic infection with higher viral loads, longer exposure to the virus, or other factors. Blood collection was performed 4 months after PCR confirmed symptomatic infection for donor B and a presumed 2–4 months after asymptomatic infection for donor A, suggesting that the timing of blood collection was not significantly different between the two donors. A correlation between neutralization titer or frequency of anti-spike expressing memory B cells and disease severity has been shown previously with large patients cohorts [36,37].

The clones were identified using the DiaSorin SARS-CoV-2 spike S1/S2 assay, but unfortunately, were not neutralizing in in vitro infection assays, using different SARS-CoV-2 isolates belonging to different clades. Our results are consistent with previous studies showing that a strong antibody response to spike protein is observed in convalescent patients, but only a small proportion of the antibodies are directed against the receptor-binding domain (RBD) and are neutralizing [36,38,39,40]. Of the neutralizing antibodies, 90% have been shown to be directed against the RBD, directly inhibiting the binding of the spike to ACE2. The other 10% of neutralizing mAbs recognize the N-terminal domain (NTD) or the stalk regions of S1 or S2 [32,33,41,42,43,44,45,46,47]. Some neutralizing antibodies may interfere with RBD or spike protein conformational changes upon ACE2 binding or with the S fusion machinery [48,49]. In addition, the induction of protein S1 shedding has been linked to neutralization potency [46,47]. RBD binding mAbs have been shown to belong most frequently to the IGHV3–53, IGHV3-66, and IGHV-1-2 families [39,50], which indeed are different from the IgHV families identified here (IGVH5-51 and 3-23).

Although the two mAbs identified here do not show neutralization activity in vitro, they may nonetheless be useful for diagnostic or other purposes, such as studies on the emerging variants of SARS-CoV-2 or other β coronaviruses [49]. Worth noting is that the role of antibodies in controlling viral infections is complex. In some cases, non-neutralizing mAbs in vitro have been shown to enhance the protection in vivo when combined with neutralizing antibodies, presumably through Fc-mediated effects [7,51]. Some antibodies may, on the contrary, favor viral spread through an antibody-dependent enhancement (ADE) mechanism. The sequences identified here are therefore being made public to facilitate future studies. Finally, the method described may be useful for smaller labs to rapidly isolate mAbs with specific functions from strongly immune donors in the context of a variety of infectious diseases, including newly emerging pathogens.

## Figures and Tables

**Table 1:** Summary of antigenic tests results.

Donor	Supernatant Tested	Spike S1/S2 IgG Test		Nucleocapsid Test	
		n. pos./n. Tested	% pos (Titer)	n. pos./n. Tested	n. pos. (Titer)
Donor A	Clones	0/91	0%	0/18	0%
	Pool	0/1	0%	0/1	0%
Donor B	Clones	2/29	6.9% 72.7 + 71.5 UA/mL *)	0/29	0%
	Pool	1/1	100% (14.9 UA/mL *)	1/1	100% (29.9 COI **)

* AU/mL: arbitrary units/mL (<12 AU/mL = non-reactive; 12–15 AU/mL = unclear; ≥15 AU/mL = reactive). ** COI: cutoff index (<1 = non-reactive; ≥1 = reactive).

**Table 2:** Deduced germline genes and level of mutation of the 2 anti-SARS-CoV-2 mAbs.

	V Gene		J Gene		D Gene
	Allele	% Identity	Allele	% Identity	Allele
MB5 heavy chain	IGHV5-51*01	96.18%	IGHJ6*02	85.48%	IGHD3-3*01
MB5 light chain	IGKV2-28*01	99.66%	IGKJ2*01	100.00%	NA
MC8 heavy chain	IGHV3-23*01	94.79%	IGHJ4*02	89.58%	IGHD1-14*01
MC8 light chain	IGKV3-11*01	98.11%	IGKJ2*01	100.00%	NA

## Supplementary Materials

The following are available online at https://www.mdpi.com/article/10.3390/antib10030026/s1, Figure S1: Nucleotide sequences of the VH and VL regions of MB5 and MC8 clones.
