# Supplementary material for: Identification of Human SARS-CoV-2 Monoclonal Antibodies from Convalescent Patients Using EBV Immortalization
Source: Antibodies (Basel). 2021 Jul 5;10(3):26. doi: 10.3390/antib10030026 (PMC8293222; doi:10.3390/antib10030026)
Supplement: Supplementary file 1 [file antibodies-10-00026-s001.zip › antibodies-1208521-supplementary.pdf]

Supplemental Fig.1. Nucleotide sequences of VH and VL regions of MB5 and MC8 clones. The sequences encoding CDR1,2 and 3 according to IMGT alignment are underlined. The codons encoding the first 4 amino acids of the constant regions are indicated in bold.

VH MB5:
gaggtgcagctggtgcagtctggagctgaggtgaaaaagcccggggagtctctgaagatctcctgtaagggttctggatacaggtttagcagctactggat
cggctgggtgcgccagaagcccgggaaaggcctggagtggattgggatcatctatcctggtgactctgataccagatatagtccgtccttccaaggccagg
tcaccatgtcagtcgacaagtctatcaacaccgcctacctgcagtggagcagcctgaaggcctcggacaccgccatgtattactgtgcgaaaaagggattt
ttggagtggttattacacggctacggtatggacgtctggggccaagggaccacggtcaccgtctcctca**gcctccaccaag**

Vk MB5:
gatattgtgatgactcagtctccactctccctgcccgtcacccctggagagccggcctccatctcctgcaggtctagtcagagcctcctgcatagtaatggat
acaactatttggattggtacctgcagaagccagggcagtctccacagctcctgatctatttgggttctaatcgggcctccggggtccctgacaggttcagtgg
cagtggatcaggcacagattatacactgaaaatcagcagagtggaggctgaggatgttggggtttattactgcatgcaagctctacaaactcctcacacttt
tggccaggggaccaagctggagatcaaa**cgaactgtggct**

VH MC8
gaggtgcagctgttggagtctgggggaggcttggtacagcctggggggtccctgagactctcctgtgcagccgctggattcacctttagcagttatgccatg
acctgggtccgccaggctccagggaaggggctggagtgggtctcagaaattagtgatagtgctggtaccacattctacgcagcctccgtgaagggccggtt
caccatctccagagacaattccaagaacactctacatctgcaaatgaacagcctgagagccgaggacacggccctatattactgtgcgaaagcgaccctg
acccggcgtgagcttgactactggggccagggaaccctggtcaccgtctcctca**gcctccaccaag**

VK MC8
gaaattgtgttgacacagtctccagccaccctgtctttgtctccaggggaaagagccaccctctcctgcagggccagtcagagtgttagcacctacttagcct
ggtaccaacagaaacctggccaggctcccaggctcctcatctatgatgtattcaagagggccactggcatcccagccaggttcagtggcagtgggtctggg
acagacttcactctcaccatcagcagcctagagcctgaagattttgcagtttattactgtcagcagcgtagtaactggcctccgtacactttggccagggga
ccaagctggagatcaaa**cgaactgtggct**
